# Wound healing: cellular mechanisms and pathological outcomes

**DOI:** 10.1098/rsob.200223

**Published:** 2020-09-30

**Authors:** Holly N. Wilkinson, Matthew J. Hardman

**Affiliations:** Centre for Atherothrombosis and Metabolic Disease, Hull York Medical School, The University of Hull, Hull HU6 7RX, United Kingdom

**Keywords:** wound healing, chronic wounds, tissue repair, diabetes, ageing, skin

## Abstract

Wound healing is a complex, dynamic process supported by a myriad of cellular events that must be tightly coordinated to efficiently repair damaged tissue. Derangement in wound-linked cellular behaviours, as occurs with diabetes and ageing, can lead to healing impairment and the formation of chronic, non-healing wounds. These wounds are a significant socioeconomic burden due to their high prevalence and recurrence. Thus, there is an urgent requirement for the improved biological and clinical understanding of the mechanisms that underpin wound repair. Here, we review the cellular basis of tissue repair and discuss how current and emerging understanding of wound pathology could inform future development of efficacious wound therapies.

## 1. Introduction

Millennia of evolution have created our skin, a highly adaptive, multifunctional organ that protects us from a daily onslaught of chemical, physical and ultraviolet radiation challenge. This harsh external environment often results in injury to the skin, and it will therefore come as no surprise that our skin possesses sophisticated reparative processes that allow it to heal quickly and efficiently. Despite considerable innate reparative ability, multiple cellular aspects of an individual's injury response can become attenuated, compromising wound closure. This attenuation is most often a result of pathological systemic changes, such as those associated with advanced age or uncontrolled diabetes. Indeed, age and diabetes are primary risk factors for developing a chronic wound (i.e. a wound that takes longer than 12 weeks to heal). Unfortunately, these chronic wounds (primarily venous ulcers, pressure sores and diabetic foot ulcers) are a major area of unmet clinical need, increasing significantly on a global scale [1]. Here, we discuss the current understanding of skin repair and illustrate impaired cellular behaviours that underpin chronic wound healing pathology. Application of emerging research technologies will be essential in further elucidating the underlying cellular and molecular basis of acute and pathological repair.

## 2. Cellular aspects of acute wound repair

**Figure 1.**
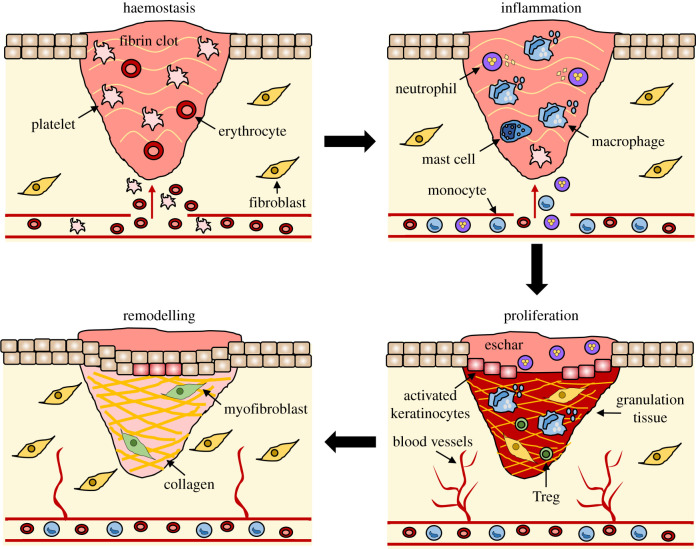
The stages of wound repair and their major cellular components. Wound repair begins with haemostasis, where a platelet plug prevents blood loss and a preliminary fibrin matrix is formed. Inflammation then ensues to remove debris and prevent infection, commencing with neutrophil influx, which is promoted by histamine release from mast cells. Monocytes arrive later and differentiate into tissue macrophages to clear remaining cell debris and neutrophils. During the proliferative phase, keratinocytes migrate to close the wound gap, blood vessels reform through angiogenesis, and fibroblasts replace the initial fibrin clot with granulation tissue. Macrophages and regulatory T cells (Tregs) are also vital for this stage of healing. Finally, the deposited matrix is remodelled further by fibroblasts, blood vessels regress and myofibroblasts cause overall wound contraction.

Our skin is specialized to interface with the external environment and provides a variety of important homeostatic functions, from regulating thermostability to sensing extrinsic stimuli. Crucially, the skin acts as a primary defence barrier, preventing desiccation and mechanical, chemical, thermal and photic damage to internal structures [2]. This defence extends to a sophisticated immune barrier response that protects against pathogenic infection, while supporting commensal microorganisms via an elegantly adapted host–microbiota axis [3]. The skin has also evolved efficient and rapid mechanisms to close breaches to its barrier in a process collectively known as the wound healing response. Wound repair is classically simplified into four main phases: haemostasis, inflammation, proliferation and dermal remodelling [4], which result in architectural and physiological restoration following damage (figure 1). The following sections describe these stages in detail.

### 2.1. Haemostasis

Immediately after injury, damaged blood vessels rapid contract and a blood clot forms preventing exsanguination from vascular damage [5]. Platelets, principle contributors to haemostasis and coagulation, are activated when they encounter the vascular subendothelial matrix. Platelet receptors (e.g. glycoprotein VI) interact with extracellular matrix (ECM) proteins (e.g. fibronectin, collagen and von Willebrand factor), promoting adherence to the blood vessel wall. Thrombin subsequently triggers platelet activation, inducing a conformational change, and release of alpha and dense granules containing bioactive molecules which reinforce coagulation (reviewed in [6]). An insoluble clot (eschar) of fibrin, fibronectin, vitronectin and thrombospondin forms [7], primarily serving to plug the wound and prevent bleeding. The eschar also fulfils a number of secondary functions, including shielding against bacterial invasion, providing a scaffold for incoming immune cells and harbouring a reservoir of cytokines and growth factors to guide the behaviour of wound cells in early repair [8].

Platelets are crucial in the recruitment of immune cells to the injury site, by either directly capturing immune cells in the eschar, or by releasing a secretome of chemokine attractants upon degranulation [6]. In fact, the platelet secretome also contains growth factors that stimulate resident skin cells, including fibroblasts and keratinocytes [9]. As the most abundant cell type during early repair, platelets play an active role in the early inhibition of bacterial infection. They express a number of toll-like receptors (TLRs) [10,11], which regulate the production of antimicrobial peptides [12]. Once a sufficient clot has formed, the coagulation process is switched off, preventing excessive thrombosis. Here, platelet aggregation is inhibited by prostacylin, thrombin inhibited by antithrombin III, and coagulation factors V and VII degraded by activated protein C [13]. At the same time, the injured vessel wall is repaired by smooth muscle cells and endothelial cells that proliferate in response to released platelet-derived growth factor (PDGF) [14]. Endothelial progenitors are also recruited to aid this process as mature endothelial cells show limited proliferative capacity [15].

### 2.2. Inflammation

Innate inflammation evolved as the primary defence against pathogenic wound invasion. This immune response is initiated by injury-induced signals; damage-associated molecular patterns (DAMPs) released by necrotic cells and damaged tissue, and pathogen-associated molecular patterns (PAMPs) from bacterial components. These PAMPs and DAMPs activate resident immune cells, such as mast cells, Langerhans cells, T cells and macrophages, by binding pattern recognition receptors to elicit downstream inflammatory pathways [16]. A subsequent release of pro-inflammatory cytokines and chemokines attracts circulating leucocytes to the site of injury (reviewed in [17]). Pro-inflammatory molecules also stimulate vasodilatation, which, along with the expression of endothelial cell adhesion molecules, such as selectins, facilitates neutrophil and monocyte adhesion and diapedesis [18]. In fact, the importance of selectins in immune cell recruitment has been clearly demonstrated, with genetic [19] and pharmacological [20] blockade of E- and P-selectin significantly impairing both immune cell infiltration and wound healing.

Neutrophils, which arrive early after injury, are recruited into the wound from damaged vessels, attracted by chemoattractants, including interleukin 1 (IL-1), tumour necrosis factor-alpha (TNF-α) and bacterial endotoxins, such as lipopolysaccharide (LPS) [21]. In response to pro-inflammatory signals, and activation of inflammatory signalling pathways (e.g. NF-κB [21]), neutrophils (and other wound cells) release their own cytokines. Neutrophils remove necrotic tissue and pathogens via phagocytosis and the release of reactive oxygen species (ROS), antimicrobial peptides, eicosanoids and proteolytic enzymes [22]. They also trap and kill pathogens in an extruded web of DNA coated with antimicrobial peptides and cytotoxic histones, termed extracellular traps [23].

The inflammatory response is complex, modulated by a multitude host of intrinsic and extrinsic factors. Uncontrolled and excessive inflammation promotes tissue injury and delays healing (as in diabetic mice [24]). However, insufficient immune cell recruitment, for example in TLR3 knockout mice, also hinders repair [25]. Thus, immune cell responses must be situational, increasing to respond appropriately to infection, yet clearing effectively to allow wound resolution. In the absence of infection, wound neutrophils decline within a few days of injury onset [26]. Most neutrophils are extruded from the wound site as they adhere to the fibrin scab, while others are removed by innate clearance mechanisms such as macrophage efferocytosis [17]. Remaining neutrophils are cleared by apoptosis, necrosis or phagocytosis, or may leave inflamed tissue and return to the circulation through reverse transendothelial migration, as observed in zebrafish [27], mice [28] and human neutrophils *in vitro* [29].

Circulating monocytes enter the wound tissue where, in response to the local milieu, they differentiate into macrophages. Although it is generally suggested that macrophages are recruited following neutrophils, an initial wave of monocytes has been observed entering the wound simultaneously with neutrophils [30]. Macrophages are master effector cells in tissue repair, displaying both versatility and high plasticity (reviewed in [31]). They reach peak wound infiltration 72 h after injury in mice and 7 days post-injury in humans [32]. Like neutrophils, macrophages engulf necrotic cellular debris and pathogenic material through evolutionarily conserved receptors, but also exhibit differential behaviours and morphological changes in response to cytokines [33].

Wound macrophages are traditionally separated into two main subsets: M1-stimulated and M2-stimulated. However, this dichotomous classification has become outdated, with both human [34] and murine [35] macrophages now known to show diverse transcriptional and phenotypic responses to different stimuli (reviewed in [36]). Hence, the macrophage repertoire should be viewed as a spectrum of phenotypes governed by tissue status and environmental signals [37,38]. For simplicity, we will herein refer to classically activated (pro-inflammatory) and alternatively activated (anti-inflammatory) groups.

Classically activated macrophages are induced by pro-inflammatory stimuli, such as LPS and interferon-gamma (IFN-γ), and promote inflammation by releasing ROS, inflammatory cytokines (e.g. IL-1, IL-6 and TNF-α) and growth factors (e.g. vascular endothelial growth factor, VEGF and PDGF). These macrophages phagocytose apoptotic neutrophils, replacing them as the main inflammatory mediator [8]. Later stages of inflammation are characterized by a transition to alternative activation, which occurs through neo-differentiation of newly recruited monocytes, or via switching of existing macrophages *in situ* to an anti-inflammatory phenotype. Although not widely characterized, this phenotypic switch can be stimulated by environmental changes in cytokines [39] and efferocytosis [40]. It may additionally be driven by miRNAs [31], transcription factors [41], and modulation of pro-inflammatory and anti-inflammatory receptors [41,42].

Alternatively activated macrophages express pro-resolutory cytokines (IL-4, IL-10, IL-13 [43,44]) and arginase, a key factor for effective wound repair [45]. Anti-inflammatory macrophages also release a myriad of growth factors to promote re-epithelialization, fibroplasia [8] and angiogenesis [46]. More recently, macrophages have been shown to be crucial in the stabilization and remodelling of blood vessels in mice and fish [47].

The importance of macrophages is further demonstrated in selective ablation studies, where *Cd11b*-specific deletion of macrophages leads to delayed wound repair and increased inflammation [48]. Similarly, inducible knockdown of macrophages during early healing caused delayed re-epithelialization, angiogenesis and granulation tissue formation, while knockdown of macrophages mid-way through healing led to endothelial cell damage, severe haemorrhage and immature granulation [49]. Thus, the collective behaviours of macrophages promote scavenging of debris, bacteria and pro-inflammatory cells, while also stimulating reparative processes to allow effective wound resolution.

The overwhelming presence of neutrophils and macrophages in wounds has potentially masked the importance of other myeloid cells in wound repair. However, recent studies have revealed that resident T cells are critical for the early injury response, while circulating T cells are recruited to resolve inflammation [50]. Indeed, aged and diabetic mice show reduced resident dendritic epidermal T cells and a delayed healing phenotype, whereas subcutaneous administration of dendritic epidermal T cells can restore healing [51,52]. Moreover, the removal of anti-inflammatory regulatory T cells delays tissue repair in mice [50]. Mast cells also play a role in wounds, releasing histamine to aid neutrophil recruitment during early inflammation [53].

### 2.3. Proliferation

The proliferative phase of healing is characterized by extensive activation of keratinocytes, fibroblasts, macrophages and endothelial cells to orchestrate wound closure, matrix deposition and angiogenesis. As early as 12 h post-injury, keratinocytes are activated by changes in mechanical tension and electrical gradients, and exposure to hydrogen peroxide, pathogens, growth factors and cytokines [54]. This activation causes keratinocytes at the wound edge to undergo partial epithelial–mesenchymal transition, where they develop a more invasive and migratory phenotype [55]. Front-to-rear polarity replaces top-to-bottom polarity, allowing the leading-edge keratinocytes to migrate laterally across the wound to reform the epidermal layer, a process termed re-epithelialization [56]. Keratinocytes behind the leading edge modulate their cell adhesion via PCKα-mediated changes in desmosome adhesiveness [57] and Eph-mediated changes in adherens junctions [58], allowing them to rearrange their order with the migrating epithelial sheet [54]. Keratinocytes in the neo-epidermis release matrix metalloproteinases (MMPs) to aid their path of migration, while laying down new ECM proteins to reconstitute the basement membrane [59].

Hair follicle stem cells are induced to proliferate, with progeny epidermal cells streaming out of the follicle to meet the cellular demand required to resurface the wound [60]. These cells sprout from damaged appendages in shallow wounds, or arrive from the epidermal edge in full-thickness wounds. Only specific stem cell compartments are activated or recruited to the re-epithelialization process [61]. For example, Krt15+ve [62] and Krt19+ve [63] bulge region stem cells appear dispensable for re-epithelialization, while Lgr5- and Lgr6-expressing cells from the follicle and interfollicular epidermis respond to wound cues, contributing to re-epithelialization [64]. A key characteristic of full-thickness wounds in mice is that appendages, including follicles, are absent from re-formed scar tissue [2]. However, under specific circumstances wound-induced follicle neo-genesis can occur, seemingly via re-activation of developmental Wnt and Shh signalling [60].

Keratinocytes negotiate through debris and necrotic tissue of the wound bed through their interactions with structural proteins of the preliminary matrix via integrin receptors [65]. MMPs, particularly MMP-1 and MMP-9, are vital for keratinocyte migration as they aid integrin receptor dissociation [56]. The production of other proteases, such as plasmin, further facilitates keratinocyte migration by degrading the provisional fibrin-rich wound bed [59]. When keratinocytes from opposing edges meet, migration terminates (via an undetermined mechanism), a thin epithelial layer is established and keratinocytes form new adhesions to the underlying matrix. Keratinocytes then fully reform the basement membrane and undergo terminal differentiation, to stratify and regenerate the epidermis [32].

Fibroblasts are the main cell type responsible for replacing the provisional fibrin-rich matrix with a more substantial granulation tissue. Resident and mesenchymally derived fibroblasts respond to a milieu of signalling molecules from platelets, endothelial cells and macrophages, including transforming growth factor (TGF-β) and PDGF. These signals direct fibroblasts to either become pro-fibrotic, laying down ECM proteins, or differentiate into myofibroblasts which drive wound contraction [55]. It is important to note that this is again a simplification, as in reality fibroblasts exhibit functional diversity, assisting dermal repair in different ways. In a seminal study Driskell *et al*. [66] demonstrated that skin fibroblasts originate from two distinct lineages, where the upper lineage aids re-epithelialization while the lower lineage contributes to ECM deposition. Recent findings have further challenged conventional understanding of wound fibroblast origin, showing that two-thirds of granulation tissue fibroblasts are actually myeloid derived [67], and are thus likely to stem from wound macrophages. Fibroblasts degrade the provisional matrix by producing MMPs and replace it with a granulation tissue rich in fibronectin, immature collagens and proteoglycans [68]. This granulation tissue acts as a scaffold for the migration and differentiation of wound cells, supporting both the formation of new blood vessels and the deposition of mature ECM.

New blood vessels are created during the process of angiogenesis to meet the metabolic demands of the highly proliferative healing tissue. Angiogenesis is triggered by hypoxia, which in turn drives the expression of hypoxia-inducible factors (HIFs) and cyclooxygenase 2, and subsequent release of VEGF and other factors [69]. In response to these changes, microvascular endothelial cells proliferate and migrate into the wound bed, sprouting new vessels that fuse with others to develop stable, tubular networks [70]. VEGF prevents endothelial cell apoptosis by upregulating anti-apoptotic proteins such as BCL-2 [71], while the fibrin matrix promotes angiogenesis by triggering phenotypic changes in endothelial cells to stimulate their migration [72].

Macrophages play a significant role in angiogenesis by aiding microvascular endothelial cell behaviours. They produce proteases such as MMPs to degrade the dense fibrin network and chemotactic factors (e.g. TNF-α, VEGF and TGF-β) to drive endothelial migration (reviewed in [73]). Willenborg *et al*. [74] demonstrated the importance of macrophage-derived factors in angiogenesis, where myeloid-specific deletion of VEGF-A reduced capillary formation in murine wounds. Macrophages also participate in the remodelling of new vasculature, by guiding vessel tips together [75], phagocytosing superfluous vessels [47,76] and dampening the angiogenic response to prevent excessive vascularization [77].

The skin houses a dense network of sensory and autonomous nerve fibres which allow sensation and movement. Nerve fibre regeneration is therefore essential following injury. Despite the principle role of diabetic skin denervation in wound pathogenesis (reviewed in [78]), wound innervation *per se* remains an understudied area. Neuropeptides, such as substance P, are known to be released from sprouting neurons and immune cells during repair, influencing diverse cellular processes (e.g. proliferation and angiogenesis [79,80]). Notably, substance P is reduced in delayed healing in diabetic wounds, where topical restoration restores healing [81,82] and contributes to nerve regeneration [83]. Wound-activated glial cells are also an important component of the repair response, shown to express factors important for chemotaxis, while the loss of glial cells delays healing in wild-type mice [84]. These and other studies suggest that innervation plays a substantial role in effective repair.

### 2.4. Matrix remodelling

Remodelling of the ECM spans the entire injury response, beginning with the initial deposition of a fibrin clot, and ending several years later with the formation of a mature, type I collagen-rich scar [55]. Fibroblasts are the major cell type responsible for wound ECM remodelling, replacing the initial fibrin clot with hyaluronan, fibronectin and proteoglycans, and forming mature collagen fibrils later in repair [85]. Proteoglycans aid construction of mature, cross-linked collagen fibrils and act as a conduit for cell migration [86]. The collagen composition of uninjured adult skin is approximately 80% collagen type I: 10% collagen type III. By contrast, granulation tissue predominantly comprises of the embryo-associated collagen type III (approx. 30%), with only 10% collagen type I [87]. As healing progresses, collagen type III is replaced by collagen type I, directly increasing the tensile strength of the forming scar [88]. The integrity and architecture of scar ECM never fully returns to that of unwounded skin. Collagen fibrils in scar dermis adopt large parallel bundles, while in uninjured skin fibrils adopt a basket weave orientation. Thus, wound scar tissue confers only up to 80% of pre-wounding strength post-injury [87,89].

These sequential changes in the ECM require a fine balance between collagen degradation and synthesis, achieved through temporal regulation of key MMPs. These collagenases, expressed by anti-inflammatory macrophages, fibroblasts and keratinocytes, cleave native helical collagens throughout repair [85]. Elastin, another key dermal ECM component, must reform elastic fibres to retain skin elasticity. Interestingly, the degradation of normal dermal matrix causes the release of elastin fragments, or elastokines, which act as signalling molecules [90]. Elastin is formed from its precursor, tropoelastin, and early in healing shows the aberrant arrangement. In fact, mature elastin fibres are often only apparent in scar tissue many months after injury [91,92].

Heightened expression of TGF-β and mechanical tension stimulate myofibroblast differentiation *in vivo* and *in vitro* [93]. Myofibroblasts are characterized by an abundance of alpha-smooth muscle actin (α-SMA), associated with an ability to generate strong contractile forces and focal adhesions [85]. Curiously, mice lacking the gene encoding α-SMA, *Acta2*, heal normally with no obvious change in fibroblast contraction [94]. This apparent redundancy, with compensation by other microfilaments, highlights the importance of wound contraction. Myofibroblast contraction is facilitated by pseudopodial extensions that allow cytoplasmic actin to bind to fibronectin in the matrix scaffold [55]. Myofibroblasts adhere to one another via desmosomes, binding to matrix fibrils and drawing the matrix together by a process termed contracture [95]. The wound healing response abates when macrophages, endothelial cells and fibroblasts undergo apoptosis or exit the injury site, leaving a scar [96].

## 3. When healing fails—factors influencing chronic wound healing

Acute wound repair is a highly dynamic cascade of cellular signalling and behavioural events that ensures rapid closure of the skin barrier. High levels of redundancy and compensatory mechanisms ensure that small alterations to this response seldom cause problems in healing wounds [97]. For example, the ablation of specific subsets of hair follicle stem cells [63], MMPs [98], fibroblast growth factors [99], TGF-α [100] and VEGFR2 [101] each individually fail to significantly impair wound closure. However, like any biological process, sufficient perturbation to the system leads to aberrations, which in the case of wounds manifest as excessive scarring at one extreme or failure to heal entirely at the other. Wounds that fail to heal (defined as generally remaining unhealed after 12 weeks) are termed chronic wounds. They primarily affect the elderly and diabetic, are highly prevalent and a major socioeconomic burden [102,103]. More effective clinical management would prevent a proportion of these wounds [104], yet many remain refractory to current treatment, highlighting the need to better understand the cellular basis of wound pathology in order to develop therapeutically viable treatments.

Susceptibility to injury remains understudied. We know that the skin of aged and diabetic mammals is more predisposed to injury, as it undergoes atrophy, with altered skin barrier and reduced hydration [105,106]. Both ageing and diabetes lead to the gradual loss of dermal matrix, with corresponding changes in tissue mechanics, loss of resilience and increased susceptibility to friction damage [107,108]. Once an injury occurs, a range of molecular and cellular perturbations contribute to overall healing impairment. One factor widely implicated in aged and diabetic wound pathology is cellular senescence (reviewed in [109]). Mitotic cells become senescent and non-proliferative in response to a host of intrinsic and extrinsic factors. Senescent cells acquire a hypersecretory phenotype, producing a secretome rich in pro-inflammatory cytokines and tissue-degrading proteases (reviewed in [110]). The chronic wound environment is the perfect platform for senescent cell induction due to the high levels of inflammation and oxidative stress [111]. Indeed, we recently demonstrated that high senescent cell burden contributes to wound pathology, where blockade of the proposed senescence receptor, CXCR2, dampens macrophage senescence and improves healing in diabetic mice [112].

A key contributor to wound pathology is excessive inflammation, which perpetuates chronicity through the continued destruction of wound tissue. Chronic wounds are characterized by high numbers of Langerhans cells [113,114], neutrophils [115], pro-inflammatory macrophages [116,117] and proteases [118–120], linked to clinical ulcer severity [121]. Along with elevated infiltration of specific immune cell subsets [122], pathological immune cell function is perturbed and collectively contributes to poor healing. Here, neutrophils are excessively primed to produce neutrophil extracellular traps, which are cytotoxic [123] and delay wound healing [124]. In diabetic mice, neutrophils are more resistant to apoptosis, and less effectively cleared by macrophages [125], furthering their excessive presence in pathological wounds. Diabetic macrophages also exhibit defective efferocytosis of apoptotic cells [126], impaired phagocytosis of bacteria [127,128] and reduced ability to polarize to an anti-inflammatory state [129]. Interestingly, even prior to ulceration, the skin of diabetic humans and mice exhibits higher numbers of mast cells and macrophages primed to the pro-inflammatory state [130]. By contrast, T cell receptor diversity [131] and the number of CD4+ T cells [116,131] are reduced in diabetic foot ulcers. Together, these aberrant features of chronic wound immune cells not only prevent the shift from inflammation to resolution, but greatly increase vulnerability to infection. Heightened inflammation may also persist due to chronic wound infection, thus maintaining the wound in a continuous cycle of infection, inflammation and inadequate repair.

Cellular impairment is not only restricted to inflammation, but also extends to re-epithelialization and dermal remodelling. Non-healing diabetic foot ulcers are typically characterized by an epidermal wound edge that is hyperkeratotic and parakeratotic [132]. Keratinocytes at the chronic wound edge show abnormal nuclear presence of β-catenin and elevated c-*myc*, which directly delays migration *in vitro* [132] and prevents healing in mice [133]. Ulcer wound edge epidermis additionally displays the misexpression of a number of cell cycle, differentiation and desmosomal markers [134], impaired growth factor receptor signalling [135], and lacks hair follicles [136]. This aberrant activation phenotype, with seemingly uncontrolled wound edge proliferation, is thought to directly inhibit keratinocyte-mediated chronic wound closure.

At the same time, dermal reconstitution is significantly inhibited by the high wound protease levels, which not only break down dermal ECM components, but also degrade growth factors (e.g. VEGF and TGF-β [137,138]) and cytokines (e.g. TNF-α [139]). Chronic wound fibroblasts are highly senescent, further compromising ECM deposition [140–142], and are unresponsive to ECM-stimulating factors such as TGF-β [143,144]. Interestingly, we recently demonstrated that deficiency in wound iron may underpin reduced ECM deposition in diabetic mice, as iron loading of fibroblasts directly stimulates ECM deposition and remodelling [145]. Macrophages are key to this reparative response, where iron sequestration causes alternatively activated macrophages to produce ECM-stimulating factors [146]. Note that disparities exist in the reported role(s) of iron in wound repair. Sindrilaru *et al*. [117] suggest that iron deposition caused delayed healing in diabetic foot ulcers, promoting an unrestrained M1-like macrophage phenotype, increased oxidative stress and senescence. Similarly, others have shown that the iron chelator, deferoxamine, improves wound healing in pressure ulcers of diabetic [147] and aged [148] mice. Thus, the cellular effects of iron are probably context-dependent and wound-type-specific, exacerbating tissue damage in an already pro-inflammatory environment, while promoting alternatively activated macrophage- and fibroblast-mediated wound resolution in late-stage repair.

Sustained hyperglycaemia in diabetes directly contributes to defective healing, compromising leucocyte function [149], inducing cellular senescence [150] and causing non-enzymatic glycation of ECM and the formation of advanced glycation end products (AGEs) [151]. AGEs not only alter the dermal structural architecture, but also trigger inflammation and ROS via their receptor, RAGE [152]. These effects impair neovascularization, in part by preventing HIF-1*α* transactivation and subsequent upregulation of VEGF and stromal-derived factor 1 (SDF-1) [153,154]. At the macroscopic level, uncontrolled diabetes causes long-term damage to the microvasculature, which results in local tissue hypoxia, arterial vasculopathy and/or lower limb neuropathy—all extreme risk factors for chronic wound development [155].

In diabetes, stem cell populations that would usually participate in vascularization are depleted (e.g. bone marrow [156]) or show impaired neovascular potential (in adipose tissue [157]). A reduction in SDF-1, which aids recruitment of endothelial progenitor cells to wounds, is also observed, while topical administration of SDF-1 accelerates diabetic wound repair [158]. Slowing AGE formation in diabetic mice improves the neovascular potential of bone marrow progenitors [159], confirming functional relevance and further demonstrating the important contribution of uncontrolled diabetes in wound pathology.

It is crucial to note that the causes of delayed healing, while simplified above, are often multifactorial and complex. Wound chronicity is influenced by local and systemic defects [160], along with imbalances in hormones, cytokines and growth factors (e.g. reduced PDGF [161]). However, in recent years, the presence and persistence of wound infection has been widely discussed as a major contributor to chronicity [162]. Indeed, high abundance of common wound pathogens, such as *Staphyloccoccus aureus* and *Pseudomonas aeruginosa*, is reported in chronic wounds [163,164], with a wound's microbial profile strongly linked to healing outcome [165]. These pathogens often develop into polymicrobial aggregates (biofilms) encapsulated in a protective matrix of extracellular polymeric substances that confers resistant to traditional antibiotics and host defences (reviewed in [166]).

**Figure 2.**
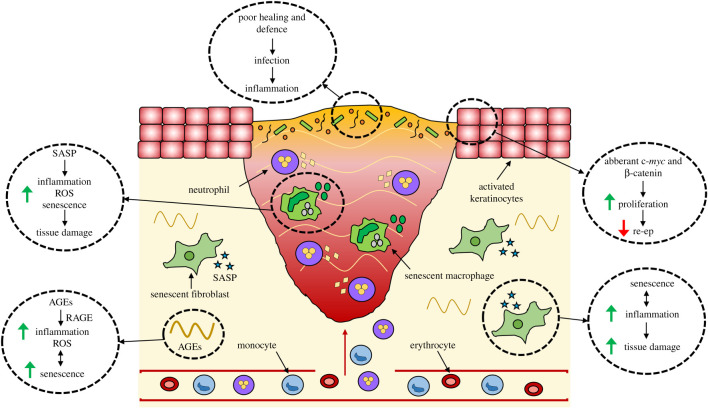
Factors contributing to chronic wound healing. Chronic wounds become infiltrated with bacteria that exacerbates inflammation. Chronic wound keratinocytes show aberrant activation causing hyperproliferation and impaired migration. A large proportion of chronic wound cells (e.g. macrophages and fibroblasts) become senescent, producing a senescence-associated secretory phenotype (SASP) that perpetuates senescence, triggers reactive oxygen species (ROS) release and heightens inflammation. High amounts of advanced glycation end products (AGEs) also contribute to inflammation and cellular senescence in the wound environment. Together these features cause excessive tissue breakdown and impair cellular functions to prevent normal healing. re-ep = re-epithelialization.

The microbiome profiles of aged and diabetic skin differ considerably from their young and non-diabetic counterparts, in each case displaying reduced α-diversity [167,168]. Although critical wound colonization occurs as a result of inadequate immune cell function, poor perfusion and the presence of a persistent open wound, it is likely (though yet to be proven) that aged and diabetic skin is intrinsically predisposed to infection by an altered microbiome. Diabetic wounds also show altered expression of pattern recognition receptors responsible for eliciting a host response, which may link to poor healing [169]. Interestingly, knockout of the pattern recognition receptor, Nod2, impaired wound closure [170] and altered the skin microbiome [171] of mice. Curiously, wild-type mice cross-fostered into Nod2−/− litters adopted an altered microbiome and acquired a delayed healing phenotype [171], therefore directly demonstrating the impact of skin microbiota dysbiosis on repair. Key factors in chronic wound pathology are summarized in figure 2.

## 4. Translational techniques to enhance clinical understanding of wounds

Our knowledge of the mechanisms underlying chronic wound healing is constantly improving, largely due to the development and refinement of wound models and diagnostic tools. For example, until the advent of sequencing technologies, wound bacterial profiling was restricted to simple culture methods, limiting speciation to only organisms capable of expansion in culture. Further analysis was then required to gather complete diagnostic information about a clinical isolate (reviewed in [172]). The emergence of short-read 16S sequencing provided new insight into clinical bacterial communities, but bacterial identification was limited to genus level based on inference from sequence homology [173], with little information about their virulence or clinical significance. Novel genomic technologies are now emerging to allow rapid molecular identification of microorganisms to the sub-species level. Simultaneous characterization of antibiotic resistance and virulence profiles [173,174] provides unprecedented insight into the role of bacterial, fungal and viral ecosystems in wound pathology. Combining these techniques with host genomic, metabolomic and proteomic approaches promises to deliver in depth understanding of the myriad of factors influencing wound repair, while ultimately facilitating a true ‘personalised medicine' approach to clinical wound management.

Historically, wound studies have relied on the use of *in vivo* models to address the complexity of the multifactorial wound response. However, it is widely accepted that between-species differences have hindered translational wound research efforts. We are now moving towards the development of more dynamic *in vitro* approaches, such as three-dimensional skin equivalents [175], allowing closer modelling of native human cell behaviours, and moving away from artificial single-cell monolayer culture. While cultured three-dimensional skin equivalents still lack many skin features, such as glands, immune cells and blood vessels, current research is beginning to address this deficit [176,177]. The development of three-dimensional-printed skin equivalents is particularly exciting, offering profound implications in translational research. Indeed, a recently developed vascularized three-dimensional-printed skin model reflected many aspects of native skin, including tissue maturation, and epidermal stratification and stemness [178].

Porcine and human *ex vivo* models are also gaining traction, with the advantage that they provide native skin tissue architecture and the full gamut of resident skin cells to recapitulate important aspects of the human chronic wound healing response [179,180]. *Ex vivo* models are not without their caveats, lacking immune cell infiltration and maintaining viability for a limited time-frame [181]. It is likely that novel culture methods, such as microfluidics [182], will extend tissue viability and allow skin perfusion with biologically relevant factors (and immune cells) to increase the relevance of *ex vivo* wound models.

*In vivo* models are still widely used, with mice favoured for mechanistic studies [183]. The multitude of available transgenic mouse lines (including reporter lines) allows temporal and spatial investigation of the molecular basis of *in vivo* wound healing. Nevertheless, strain- and species-specific differences must be considered, especially when extrapolating conclusions for translational research purposes. Pigs, though used far less frequently, provide a useful translational model with skin that closely resembles that of humans. Wounding in mice involves full-thickness incisions or excisions, yet variability can be introduced between laboratories by the methods used to apply wounds, the analgesics and anaesthetics used, and how the wounds are treated (e.g. splinted, occluded or left to heal by secondary intention [184,185]). Continued efforts to standardize *in vivo* methodology will be essential to increase experimental validity and progress current and future wound research.

An array of pre-clinical delayed healing models are used to better recapitulate human chronic wounds, from pressure ulcers in mice using magnets [186], to infected wounds in pigs [187]. As those primarily at risk of developing chronic wounds are elderly or diabetic, it follows that the most widely used chronic healing models involve aged and diabetic rodents [188]. Type I and type II diabetes mellitus (T1DM and T2DM) can be modelled in mice. T1DM-mediated delayed healing is commonly stimulated through streptozocin injection [189,190], where timing post-injection is critical to the delayed healing phenotype [192]. Genetically altered mice are used to mimic T2DM through leptin or leptin receptor deficiency. These mice are morbidly obese by 6–8 weeks of age, go on to show hallmarks of T2DM (reviewed in [193]), and display substantially delayed healing versus their non-diabetic, heterozygous littermates [194]. There remains some controversy as to whether delayed healing in diabetic mice is a result of hyperglycaemia, leptin deficiency or obesity [184].

To mimic age-associated healing pathology, mice are wounded at 18 plus months of age (reviewed in [195]). Young ovariectomized mice provide an alternative accelerated ageing model, where surgical removal of the ovaries mimics the human menopause [196]. Here, the loss of circulating sex hormones, particularly 17β-estradiol, produces a delayed healing phenotype that is largely comparable to that of aged mice (reviewed in [197]). Unlike diabetic models, limited to comparison against diabetic wounds, aged models have the advantage that they emulate a more generalized underlying risk factor for all chronic wounds, advanced age [198].

## 5. Current therapies and future opportunities

Wound management begins with an assessment of wound aetiology and a patient-centric approach to managing systemic and lifestyle factors. In the case of diabetic foot ulcers, local management often starts with debridement, the removal of necrotic, infected or hyperkeratotic tissue via surgical or less invasive modalities [5,199]. Extracting the chronic tissue back to less affected epidermis, while triggering an acute injury response, is thought to kick-start normal reparative healing pathways [200]. Wounds are then irrigated with saline or antibacterial solution and a tailored dressing is applied [201]. Contemporary dressings contain a myriad of material properties to aid tissue repair and incorporate substances with known pro-healing or antimicrobial effects [202,203]. More advanced solutions are available, including the continually evolving negative pressure wound therapy modality [204]. Despite numerous available treatments, current best practice wound management is almost exclusively aimed at addressing secondary causes of chronicity, while also relying heavily on patient compliance. These two factors result in up to 40% of chronic wounds persisting for many months or years despite extensive treatment [102]. There remains a clinical unmet need to address this shortfall with novel therapies that are financially, physiologically and practically viable for the wound care setting.

A major contributor to chronic wound recalcitrance is persistent, antibiotic-resistant biofilm infection. It is therefore unsurprising that a large proportion of recent wound research has focused on the development of novel antimicrobial and anti-biofilm therapies. Traditional non-antibiotic antimicrobials, such as silver salts, alleviate bacterial burden but are cytotoxic to the host, while modern formulations (e.g. nanoparticles) have lower cytotoxicity and may also promote wound healing (reviewed in [205]). Emerging antimicrobial treatments that may also show beneficial roles in tissue repair include cold atmospheric plasma [206,207] and bioactive glass [179,208].

Most antimicrobials display broad effects and are not targeted to specific pathogenic species and strains. This is important, as commensal bacteria have a positive role in skin maintenance and wound repair (reviewed in [209]), and unlike their pathogenic counterparts, commensal biofilms do not cause persistent delayed healing in diabetic wounds [166]. As a result, more directed treatments for pathogenic bacteria, such as phage therapy [210] or pharmacological inhibition of bacterial virulence mechanisms such as quorum sensing [211], may confer higher specificity and efficacy. Moreover, most treatments focus on the bacterial component of infection, but the fungal diversity of wounds is also linked to healing outcome [212]. Thus, to elucidate the role of host–microorganism interactions in pathological repair, prospective research should acknowledge the wound ecosystem in its entirety.

**Figure 3.**
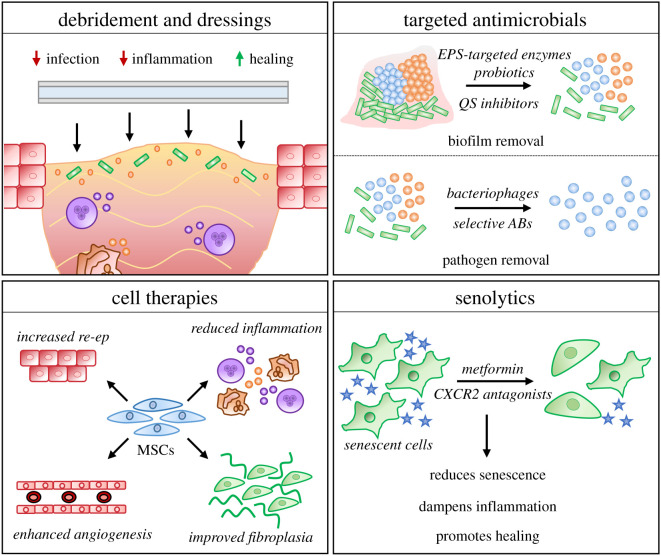
Traditional and novel chronic wound treatments and their major tissue effects. Debridement of infected and necrotic tissue, followed by tailored dressing use, is common in wound treatment, with the aim of reducing microbial burden, dampening inflammation and providing a more suitable environment for healing. Antimicrobial therapies are emerging to disrupt biofilms and selectively remove pathogenic, rather than commensal, organisms. EPS = extracellular polymeric substance. QS = quorum sensing. ABs = antibiotics. Cell therapies such as mesenchymal stem cells (MSCs) can benefit multiple aspects of wound repair. re-ep = re-epithelialization. Finally, targeting chronic wound senescence with senolytics (e.g. metformin or CXCR2 antagonists) may be a viable option to reduce inflammation and promote healing.

Experimental studies are providing new insight into the underlying molecular and cellular correlates to chronic wound pathology. This in turn offers exciting new avenues for future therapeutic prevention and intervention. For example, chronic wounds are burdened by high levels of cellular senescence [141,142]. Senolytic drugs such as quercetin target senescent cells, and have already shown promise in reducing senescent cell burden in pathology [213,214] and ameliorating symptoms of diabetes, including inflammation and hyperglycaemia (reviewed in [215]). Further, blockade of the senescence-linked receptor, CXCR2, directly accelerates diabetic wound repair *in vivo* [112]. Repurposing these existing treatments (a number of senolytics drugs and CXCR2 antagonists have been tested in clinical trials [216,217]) offers an attractive approach for wound management. Other cell-targeted strategies include the administration of stem cells (reviewed in [218]), growth factors (reviewed in [219]) and gene therapies (reviewed in [220]). The major reparative effects of emerging and potential chronic wound therapies are outlined in figure 3.

## 6. Conclusion

The high cellular diversity, complexity and plasticity of wound healing provide a considerable challenge to comprehensively elucidate. While this remains a perplexing goal, it is essential that we continue to strive to more fully understand the mechanisms that underpin both normal and pathological healing. While not without their limitations, emerging wound models provide an unprecedented opportunity to further explore the molecular and cellular features of wound repair. Combining these approaches with novel tissue, cell and molecular ‘omics' technologies will considerably advance our understanding of wound pathology. Indeed, the future holds great promise for the development of innovative new therapeutic strategies for advanced wound care.

## Data accessibility

This article has no additional data.
